# Participatory Design With Seniors: Design of Future Services and Iterative Refinements of Interactive eHealth Services for Old Citizens

**DOI:** 10.2196/med20.2729

**Published:** 2013-10-08

**Authors:** Isabella Scandurra, Marie Sjölinder

**Affiliations:** ^1^Division of Visual Information and InteractionDepartment of Information TechnologyUppsala UniversityUppsalaSweden; ^2^SICS Swedish ICTSICSStockholmSweden

**Keywords:** Internet, community-based participatory research, systems analysis, interdisciplinary communication, community networks, professional-patient relations, seniors, elderly users, television, social inclusion

## Abstract

**Background:**

There is an increasing social isolation among the elderly today. This will be an even larger issue in the future with growing numbers of elderly and less resources, for example, in terms of economy and staff. Loneliness and social isolation can, however, be addressed in several ways using different interactive eHealth services.

**Objective:**

This case study investigated novel eHealth services for the elderly, and their usage of a social interactive device designed especially for them.

**Methods:**

In this work, we used an innovative mobile communication device connected to the television (TV), which worked as a remotely controlled large interactive screen. The device was tested by 8 volunteers who visited a senior center. They were between 65 and 80 years of age and lived in their own homes. Throughout the 1.5 year-long project, 7 design workshops were held with the seniors and the staff at the center. During these workshops, demands and preferences regarding existing and new services were gathered. At the end of the project the participants’ experience of the device and of the services was elaborated in 3 workshops to get ideas for improved or new meaningful services. During the data analyses and development process, what seniors thought would be useful in relation to what was feasible was prioritized by the development company.

**Results:**

Regarding daily usage, the seniors reported that they mainly used the service for receiving information from the senior center and for communication with other participants in the group or with younger relatives. They also read information about events at the senior center and they liked to perform a weekly sent out workout exercise. Further, they played games such as Memory and Sudoku using the device. The service development focused on three categories of services: cognitive activities, social activities, and physical activities. A cognitive activity service that would be meaningful to develop was a game for practicing working memory. In the social activities category, the seniors wanted different quizzes and multi-player games. For physical activities, the seniors desired more workout exercises and suggestions for guided walking routes. A new category, “information and news”, was suggested since they lacked services like senior-customized global and local news.

**Conclusions:**

This study showed the importance of input from a group of seniors when designing new services for elderly citizens. Besides input to interactive eHealth service development for seniors, this study showed the importance of a social context around such work. The seniors were very engaged throughout the project and workshops were frequently visited and the seniors became friends. The high amount of input from the seniors could be explained in terms of social inclusion; they belonged to a group and each member was considered important for the work. The friendly workshop atmosphere facilitated new ideas and redesign of the services.

## Introduction

### Background

There is increasing social isolation among the elderly today. Loneliness and social isolation can, however, be addressed in several ways using different interactive eHealth services. One of the most important issues in reducing social isolation is the existence of social networks [1]. The possibilities to communicate with friends and relatives through computers and the Internet can increase the social network, and social isolation can be reduced [1,2]. Several studies have shown the importance for older adults to be able to communicate with family members and friends enabled through new communication technology [2,3]. Although it may seem different, the Internet usage pattern does not differ between older and younger daily users; Internet is mostly used for emailing, searching news, and gathering practical information [3]. This technology also provides opportunities for older adults to gain new knowledge from other generations. The possibility to communicate with children and grandchildren through the Internet and email, for example, is important for older adults and has already become one of the most important reasons for older adults to purchase a computer at the end of the last century [4]. Older adults using the Internet have also reported that they experience a higher level of social support [5]. Among many elderly, Internet has also become an important source for getting medical information. This opportunity increases the feeling of controlling the own health. It empowers people in terms of being more educated in the area, and having the possibility to share experiences with others [6].

The quick development in the area of mobile communication in the last decade has provided many new possibilities for communication and sharing our everyday life with each other. Many new services and applications are also targeted towards the elderly and have interfaces that are considered easy to use. In Sweden, however, where Internet access is common and accessible almost everywhere, daily usage decreased from generation to generation as a function age (Table 1).

Table 1 clearly reveals that Internet use decreases with increasing age. These data show as well a longitudinal measure of three user groups. Following a specific group of seniors horizontally, it is noticeable that daily use of Internet increased from 2009 to 2011. In 2011, the daily Internet use was 51% for the age group 65-74 years old and 22% for those 75 years old and up, which was the oldest measured group [7].

The low rate of usage at high ages could become a society problem, where more and more public services are accessed via different communication technology tools. This is also valid for eHealth services, where for example, time booking and contact information to care providers, as well as access to own health and social care data is found on the Internet and difficult to access elsewhere. It is therefore imperative to develop user- and situation based eHealth services that are thoroughly tested with and accepted by the intended users.

**Table 1:** Daily Internet use in Sweden during three years and displayed in three groups of users [7].

Age group	2009	2010	2011
55-64 years old, %	53	57	64
65-74 years old, %	37	43	51
75 years old and up, %	12	16	22

### Objective

This case study aimed to investigate novel eHealth services for elderly citizens together with seniors, using an interactive device designed for seniors.

### The Project: “Quadruple Helix”

This study was set for 1.5 years, financed by the Swedish agency for innovation systems, Vinnova. The goal with the project was to jointly develop a range of services that correspond to the society’s need for new sustainable and quality assured services in elderly care. The work in this project can be described as a kind of innovation procurement in which municipalities raise their competence, and where the Information Technology (IT) development companies increase their knowledge of the end users. By collaborating with researchers, the company gets a chance to strengthen their methodological approaches. By involving seniors, the project model is raised from being a Triple Helix (society working closely with industry and academia) to an even stronger quadruple helix model with the main stakeholder actively involved as potential end users [8]. In this environment, problem-owners, health informatics researchers, elderly users, and developers of novel IT services worked together in a user-centered and participatory design approach.

### Research Approach

This research study adheres to cooperative design [9,10], which is a human-computer interaction (HCI) research theory that regards system development with user participation and considers designing a social process. From research literature, we know that usability aspects should be brought in early in the development process [9,11]. Previous research also presents several methods to engage users in the future, for example, future workshops [12]. Other methods to bring future needs analysis into system development are iterative prototyping and scenario-based design, preferably applied together with potential users in a collaborative approach [13,10].

The degree of user participation may vary. Regardless of activity degree, in cooperative design developers and practitioners/users are seen as actively cooperating partners. Together they aim to reduce uncertainty and risk in the development of novel systems, where a detailed conception of exactly which future needs should be supported, is often lacking [9,12]. Moreover, using older people’s extensive experience when trying to meet their needs can be more successful for promoting a new product or service, rather than relying on interaction patterns based on the computer paradigm [14]. This fact increased the interest to further study how novel eHealth services could be designed to reach a heterogeneous target group of elderly people, spreading over an entire country, across different ages, education, health status, and interests. This case study is one of four aiming to extract new knowledge based on user experiences of different older citizen groups using this device and testing its interactive eHealth services [8]. It was conducted together with seniors healthy enough to frequently visit a senior center.

##  Methods

### Device and Test Participants

**Figure 1:**
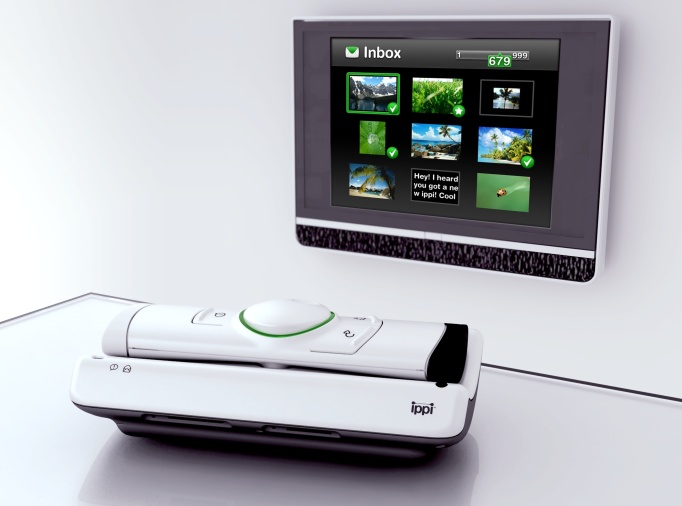
The communication device, a prototype called ippi, connected to the TV-set.

Research suggests that use of a TV as the platform would reduce new users’ uncertainty [15,2]. Based on this previous research, the TV platform was believed to have a relative advantage over computers and mobile phones in terms of users’ self-reported motivations for starting and continuing to use the system. Hence, this study used an innovative mobile communication device (Figure 1) connected to the TV, which worked as an interactive large screen controlled remotely.

When the device was connected to an ordinary TV, the TV could receive and send photos, videos, sounds, and text messages from mobile phones and computers. The technology behind this was based on the mobile phone network for communication. There was a subscriber identity module (SIM) card in the device, requiring the device to be placed within global system for mobile communications (GSM) coverage. It was also possible to send email through the device.

The device was considered easy to install in the home. It was plugged into the TV and to the power connector. When a message has been received, the device flashed like an answering machine. The message could be opened with one press on the main button of the remote control. The user of the device could answer the message by writing a text or by sending a voice message. The device could be used for communication between friends and relatives, but also for caregiving purposes, for example to inform the senior which nurse from social care service was scheduled to come, the task to be performed, or medication to be taken.

This prototype, not widely commercialized yet, has been iteratively developed over the last three years. The studied (and latest) version consisted of three different user modes addressing different user groups. User mode 1 aimed to address the basic needs of the elderly without technology experience living at nursing homes. User mode 2 was targeted to a more active user group that still lived on their own, but with nursing or home help support. Some of the most advanced functionalities had been closed to make the device easy to use for people without technology experience. The aim with user mode 3 was to provide full possibilities for mobile communication (text messages, email, sending pictures, etc) for a cognitively active senior living on their own, with or without support from the municipality. These target groups were handled in different studies.

The participants were not randomly selected from the intended user population. Instead, the recruitment process of the participants of this study was handled by the senior center, following the stated requirements: the seniors should be capable of using the functionalities of a computer or a mobile phone, but for different reasons not wanting to communicate through these devices. Some technological skills and experiences were required as this participant group was selected to provide the development company with as much input as possible to the design phase of new services. A total of 8 seniors volunteered and fitted the description of user mode 3. They were between 65 and 80 years of age and lived alone in their own homes. The seniors were not acquainted, but they lived in the neighborhood of the senior center and had visited the center previously, where the advertisement for this project was posted. They all volunteered to test the services and they consented to participate in the study.

### The Study

#### Setup

The participants were recruited by the senior center where an information booklet about the project and one device had been placed. Interested seniors could read about the project and try out the device before deciding to participate. Before the participants were given a device to bring home, they were offered introductory information and an education meeting aiming to create comfort and curiosity of the device and its services. Each participant was given a device, installed it at home, and started to use it with full access to all functionality. There was also one device at the senior center that was used when the group met at the center. The staff at the senior center also used their device for communication with the senior user group.

Most of the seniors installed the device at home by themselves. The ones that needed help had plugs on the TV set in places that were difficult to reach. At the beginning, support was given by staff from the senior center. Second line support was given by the development company during the entire project.

Throughout the 1.5 year-long project, 10 evaluation workshops were held with the seniors and the staff at the center. Two development workshops were held with the development company and researchers. Formative qualitative evaluations consisted of two parts:

1. Design workshops (n=7) to evaluate existing services and suggest improved functionality of the device and interaction with the services.
2. Future workshops (n=3) to get user contributions for the design of new meaningful services.

#### Part 1: Design Workshops

The seven design workshops were held approximately once a month and lasted for two hours including a coffee break. Seniors, researchers, staff from the senior center, and representatives from the developing company participated. The design workshops were entirely user-centered and the main goal was to gather the seniors’ demands for improved functionality and interaction with respect to existing services. The seniors and the staff were very active during the workshops, where a large focus was placed on the participants’ questions and demands regarding the device and its services.

All workshop occasions started with a discussion where the participants had the opportunity to ask questions about the device and its usage. Feedback was given by the developers both by explanations and on-site education of existing services and by iteratively shown refinements in new demos of the services. Improvements related to ease of use were accepted by the senior group after individual hands-on testing.

The participants also used questionnaires to describe daily usage of the provided services. The questions covered topics such as how they had used the device, who/how many people they communicated with through the device and what they had learned since last meeting. These questions were walked through during the workshop and all participants answered or commented from their perspective. During the discussions, one researcher took notes and wrote down the participants’ answers and other issues that evolved in the discussion. At each workshop, data gathered in the previous workshop was discussed. The aim was to establish a correct understanding of the participants’ ideas and issues. At the end of the design sessions a longer questionnaire was filled in. It gathered information about the participant’s present usage and bridged to the desired future usage. The questionnaire also contained questions about how often/when they used the device, about attitudes towards usage (eg, if it was fun to use the services and why).

The main goal with the workshops was to gather user information in order to improve the device and its functionality. The discussions did not contain sensitive or personal questions, so we are rather persuaded that the participants shared their thoughts with the only intention to improve the product. Many suggestions for improvements were given, and it is not likely that biases like the “Hawthorne effect” played a major role. On the contrary, the focus was on improvement of a novel device and the users were aware of that their mistakes and misunderstandings were valuable information for the developers. The frequent meetings also contributed to an atmosphere were the participants felt comfortable when being negative towards the technology.

The approach was qualitative and analyses were inspired by the constructivist grounded theory method [16]. The material was analyzed and coded based on gathered notes and written answers from the participants. Concepts were interpreted and categorized by the researchers and handed over as summarized improvement opportunities to the developers.

#### Part 2: Future Workshops

##### Overview

In the last three workshops, the main focus was to move from improvements of existing services to the design of new communication or social inclusion services.

The future workshops were conducted to cover the process from user requirements to prioritization of suggested services by the seniors. They consisted of five phases, performed with different participant groups, both with seniors and with the project management group. To visualize new services for other users than “myself” more easily, the seniors were instructed to create three “personas” [17], which they later used as representations of other types of seniors.

##### Phase 1: Future Workshop at the Senior Center

The first phase consisted of a brainstorming session on how to use the device in the future using post-it notes with seniors and the project team. The only limitation of the exercise was that the proposed services should fall within the following areas: cognitive activities, social activities, and/or physical activities.

##### Phase 2: Categorization and Detailing of Future Workshop Material

The material from phase one was categorized and subgroups of services were created. Where needed, details were added to concretize the services. The categorization was performed by the project management team and led to over 50 proposals for various new services based on the suggestions from the seniors.

##### Phase 3: Feasibility Prioritization

The third phase consisted of a project in-house seminar on project priorities among suggested services with a focus on feasibility, with respect to content and cost.

##### Phase 4: Future Services Prioritization Based on Potential Value for the Seniors

The fourth phase regarded prioritization by the seniors. Based on the 17 services that passed phase 3, the purpose was to let seniors choose the services that they considered most valuable to realize.

##### Phase 5: Concluding Validation Workshop

As the last phase of the future workshops, both participants of the workshop series and other senior stakeholders were invited to prioritize amongst the 17 services left. The results of this concluding validation workshop are described below.

##  Results

### Part 1: Design Workshops

#### Usage

The device itself was tuned during the workshop period, and the users considered it “stepwise more easy to use”, both regarding functionality of the device and the interaction with the services. Regarding daily usage, the seniors reported that they mainly used the service of receiving and replying to invitations of events from the senior center. The staff at the center sent out a schedule for each week and the messages provided a direct contact with the seniors. This also helped the center to plan and improve publicly given events.

Furthermore, the participants used the device for communication with other study participants or with younger relatives. They also played games as Memory and Sudoku, and they liked to answer quizzes. Finally, the seniors enjoyed performing the weekly-distributed workout exercises.

Both the weekly workout exercises and the quizzes were services that were introduced and tested during the project. These services were developed in an iterative way based on suggestions from the seniors, aiming to encourage individual physical and cognitive wellness.

#### To Increase Future Usage

The seniors desired multiple services that they found attractive, in order to use the device more often. The hardware of the device was criticized by some users as being too old-fashioned. The text input mode was too cumbersome and the navigation within and between the services could be made more explicit. Finally, the participants pointed out the importance of keeping intact the already existing possibilities to communicate with children, grandchildren, and friends.

#### Attitudes Towards Acceptance and Usage

One of the questionnaires regarded the seniors’ attitudes towards using the device and its services (Table 2). The questions were inspired by factors known to contribute to acceptance of an innovation [15]. The questionnaire used a numeric scale and the results are presented in a descriptive statistical manner. The generated results are not generalizable as such, instead they can be regarded as indicators of how the device was accepted and used by these novel users.

The results regarding attitudes towards acceptance and usage showed that the seniors thought it was quite easy to use the device and they managed to perform the actions they wanted to do. However, this could to some extent be explained by the frequent sessions at the senior center were they could get support. Nevertheless, a positive result was that the device was used and that the seniors felt that it fulfilled its purpose. Finally there were questions regarding to which extent they talked to others about the device and their usage. The aim with these questions was to understand whether the seniors were proud of having this device. The answers here were highly rated (4 of 5) and a conclusion could be that the seniors felt they had access to something new and useful, and that being a part of this development process was something they wanted to tell other people about.

**Table 2:** Attitudes towards using the device and its services.

Question	Scale: 1=low ; 5=high	Rating
Degree of fun to use the device	1=Not fun at all; 5=Very fun	3.3
Degree of easy/hard to use the device	1=Very easy; 5=Very hard	1.7
Degree of success in doing what they wanted to do with the device	1=Not at all; 5=To a great extent	4.0
Degree of telling others that they had this device	1=not at all; 5=To a great extent	4.0
Degree of telling others why/ how they used the device	1=not at all; 5=To a great extent	3.3

### Part 2: Future Workshops

The requirement list of new future services resulted in improvements of three categories of services: cognitive, social, and physical activities. Desired future services contained news in general but in particular local news, about events to happen, or recaps of happenings. Therefore the participants also invented a new category “information and news”, since they lacked senior-customized global and local news services, as well as municipality information about local events like lectures or cultural events, suitable for seniors. The entire future workshop process resulted in the high-priority proposals highlighted in Table 3.

**Table 3:** High-priority proposals from the future workshops.

Category	Details
Cognitive activities	Memory matrix: a game for practicing the working memory
Social activities	Quizzes with stepwise clues (like in magazines)
	Different multi-player games: to compare yourself with other users’ results
	Cooking tips: daily or weekly menus.
Physical activities	Low impact workout: gymnastic exercises for seniors/elderly (Figure 2)
	Guided walking routes: discover your neighborhood
Information and news	Senior-customized news from the municipality (or other service providers)

**Figure 2:**
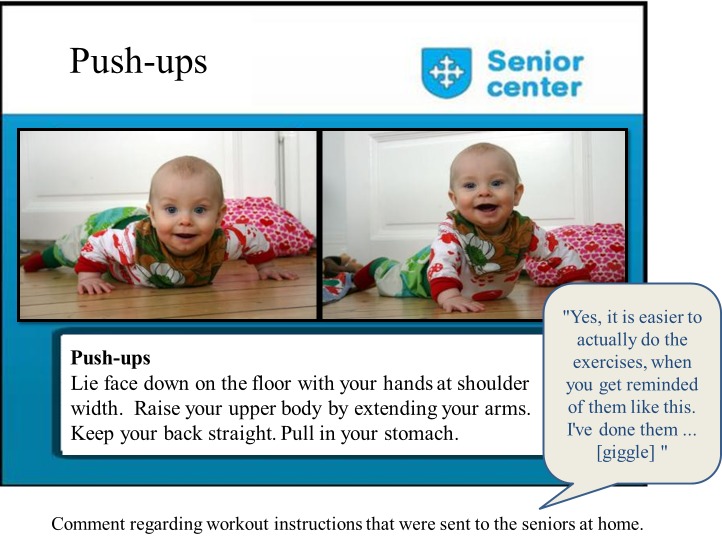
An example of a workout instruction for seniors; push-ups. This kind of illustration of the workout exercises (using a baby instead of an adult) was appreciated by the seniors.

##  Discussion

### Principal Findings

To summarize the results regarding daily usage, the seniors reported that they used the service for communication with the participants in the group or with younger relatives. This is in line with previous results showing that the use of information technology increases social interaction [1,2]. The seniors also reported that they used the service for receiving information from the senior center, and to read information about events at the senior center. Furthermore they liked to perform a weekly-distributed workout exercise, and they played games such as Memory and Sudoku using the device. The service development focused on three categories of services: cognitive activities, social activities, and physical activities. The material was prioritized based on the seniors’ ideas of usefulness and the developers’ feasibility studies. A cognitive activity service, found meaningful to develop, was a game for practicing the working memory. In the social activities category the seniors wanted different quizzes and multi-player games. For physical activities the seniors desired more workout exercises and suggestions for guided walking routes. A new category “information and news” was suggested since they lacked services like senior-customized global and local news.

### Reflections Regarding the Study Setup

As already mentioned, the participants iteratively filled in questionnaires during the project. At the concluding workshop the participants described their overall experiences, both with respect to the latest version of the device and with respect to the overall impressions about the project.

Hands-on work with innovations is never easy and often time-consuming in the start of a user-centered project, but it pays off in the long run if the user feedback is thoroughly handled. The group format with the senior participants was a good method to learn about what worked and how to improve the intervention. In each workshop the users’ reflections were gathered and brought into the development process. Both this study and other research [14,18] showed the importance and the benefits of using older people’s knowledge and experience in the development of new products.

Developers from the company participated in all workshops with the seniors. When working closely with researchers and users, the developers improved their understanding of the potential users and the real usage context. In this case the researchers’ work was to support direct communication between users and developers rather than gather material to hand over to the non-present developers. If the developers would not be present at the workshops, much explanation time would be consumed and a real understanding for the users’ needs and preferences could be lost.

It is imperative to let the refining and detailing work of the services take its time in the project management group (developers and researchers) in order to avoid misunderstandings, based on different views of what the seniors actually desired. It is difficult to put one’s technical skills aside to fully understand the needs and wishes of so-called non-technical users. Feedback from the seniors was necessary to ensure that their needs were properly understood, as well as the need of an iterative development method to handle suggestions of improvements and new services. During the project, new services such as workout exercises, memory training, and quizzes were implemented thanks to the demands from the seniors.

The seniors’ usage of the device and the services were also practical hands-on work. They had an own device at home and access to the device at the senior center for learning purposes, either together with the staff or with other participants in the group. In short, the seniors had access to both the technology any time they wanted at home, and several channels to get support. We are persuaded, that having access to the technology at any time, the possibility to get support easily, and frequent sessions with the developers are most important aspects in successful technology development.

Furthermore, it is crucial to have frequent access to the technology to be able to integrate the usage in one’s life [15,5]. Only when the technology is used in a realistic way, based on specific needs, will useful and correct feedback be given [18]. The frequent meetings also provided the possibility to get to know each other and created an environment where all participants in the quadruple helix-team could suggest changes freely. It was evident that this team was working hard together towards a common goal to improve the device as much as possible.

It was also evident that a novel device cannot be perfect from the beginning. It has to be iteratively refined, tested by relevant user groups, and evaluated by future users. Often technology can be rejected based on a tiny aspect. Good communication with future users and usage observations makes it easier to detect these aspects and remove potential problems. A benefit of the work in a quadruple helix-constellation like this is that the developers get a deeper understanding of why certain changes need to be performed to reach good usability of a product.

### Social Inclusion

This work provided input to the development of different social services and it showed increased social contacts, especially with grandchildren. Besides input to the design of new services, for example providing local information and support for physical activity, this work showed the importance of a social context around such a study. The seniors were very engaged throughout the project. The amount of input from the seniors could be explained in terms of social inclusion; they belonged to a group and each member was considered important for the work. The friendly workshop atmosphere facilitated new ideas and the work with redesigning the services.

Between the monthly held workshops, the seniors decided to meet on their own for doing their “homework” and to share new knowledge. The seniors appreciated that they could learn the technology together with other participants who were at the same level. The user group has also been welded together socially and, thanks to the project, they now socialize privately and agree on having made new friends.

The perception among the seniors was that it was exciting to participate in a process where new technology was designed and developed. Participation also seems to have opened up the participants' interest in technology beyond this device and its services. One of the participants believed that she had become more confident to handle the DVD to the TV, a spillover effect of daring and learning new things within this project.

The importance of active participation in the society, even without using a computer, has also been identified in the project. A senior got a job via the device because she had an email address through which she was contacted. Using an email address connected to the TV, non-computer users are also represented in the digital world. Regarding development of digital cultures, there is a possibility to decrease the amount of “socially and technically excluded” (no access available) or “expelled” (forced to live without Internet) groups of people, as defined by Wyatt [19], just by providing devices like the one tested in this study.

### Future Work

In this project we gained insight on a number of positive social aspects. Some were planned for and others not. One future interesting design aspect to be further investigated is how social benefits (and other benefits) can be used in the design process in a more structured way, and how the process itself can fulfill human needs. This will in the end also lead to even more engaged participants managing to provide improved input to the design process.

We have earlier been working with similar settings in other case studies (eg, [13]) and again, we noticed how valuable the researchers’ mediation is during communication between different stakeholders. The translation or mediating activities create a mutual understanding. An interesting area to be further investigated is how the direct communication between participants and developers can be taken one step further.

We would also like to study how the method for prioritization could be refined. In this work we had a number of services that the users rated on a usefulness scale and the developers on a feasibility scale. The results were put in a diagram with two axes. We believe that this process/method could be further developed towards an efficient tool for service development and evaluation.

Another interesting task will be to ensure creation of win-win situations in the beginning of a quadruple helix project. It is evident when a project like this ends, that it has increased knowledge in the various stakeholders’ organizations. However, being able to actually measure project goals related to the benefit and win-win situations is rare as that kind of project goals often lack in a project plan. Stated and measurable win-win situations and explicit benefit would probably aid when spreading this method further.

The work performed in this study, together with the other three studies in this project, tried to define potential users based on specific user requirements for this novel device [8]. It would be interesting to test this device in another context, for example, in stroke patients’ care and rehabilitation. In rehabilitation, it is common that elderly patients’ contacts with caregivers after some time become less frequent. Both parties suffer when they receive too little information and consequently proper follow-ups are missed [20]. New channels of receiving information and new ways to communicate will probably increase the possibilities for elderly to be more active in their own care and rehabilitation.

### Concluding Remarks

The aim with the project was to develop services and functionalities that meet the needs among seniors, both today and in the future. Throughout the project new services have been introduced with the purpose of being useful for seniors or elderly people living in their own homes. Based on a user-centered approach and an iterative development process, the services and the functionalities were a result of this specific context, with this group of seniors, developers, and researchers. The outcome was specific for this context and hence, the results are not generalizable. However, use of personas and a careful recruitment process seeking to find representatives of potential future users is in line with previous research [10,17]. Therefore it is fair to claim that the results can be regarded as important indicators of a useful development approach where the aim is to develop technology that is of instant benefit for the user group. The local recruitment of seniors that were interested in the device also placed a focus on the motivational aspects. One explanation to the participants’ engagement is that they were individuals that found this task relevant and interesting. People are different and have different needs; we believe it is important to develop technology with and for people that find the services meaningful rather than trying to develop towards specific target groups, without their engagement in the development process.

Another important aspect in the development is to actually understand the context around the users and make sure that the developers understand this context as well. Our results seem to be cost-effective when developers meet groups of users rather than getting second-hand information. When the developers are a part of the workshop, a source for rich information about context and needs arises that can only be conveyed by similar approaches. Participation makes it easier to develop the right product from start, or at least getting as close as possible.

To summarize the successful outcome of the project, the seniors were given a number of new, essential, services as described above. Most important from a socio-technical development perspective is that the participants enjoyed using the device, they participated to further develop it and they wanted to continue using it after the end of the project.

## Abbreviations

GSM: global system for mobile communications
HCI: human-computer interaction
IT: information technology
SIM: subscriber identity module
TV: television
